# Silicon Nitride Bioceramics Sintered by Microwave Exhibit Excellent Mechanical Properties, Cytocompatibility *In Vitro*, and Anti-Bacterial Properties

**DOI:** 10.3390/jfb14110552

**Published:** 2023-11-17

**Authors:** Jiayu He, Yuandong Liu, Xiaofeng Zeng, Yan Tong, Run Liu, Kan Wang, Xiangdong Shangguan, Guanzhou Qiu, Coswald Stephen Sipaut

**Affiliations:** 1 Key Laboratory of Biohydrometallurgy of Ministry of Education, School of Minerals Processing and Bioengineering, Central South University, Changsha 410083, China; 215611005@csu.edu.cn (J.H.); 235612137@csu.edu.cn (Y.T.); 202208600021012@ctgu.edu.cn (R.L.); 225611016@csu.edu.cn (K.W.); 215612085@csu.edu.cn (X.S.); qgz@mail.csu.edu.cn (G.Q.); 2 Hengyang Kaixin Special Material Technology Co., Ltd., Hengyang 421200, China; jishuyfdept@hykaixin.com; 3 Faculty of Engineering, University Malaysia Sabah, Kota Kinabalu 88400, Malaysia; css@ums.edu.my

**Keywords:** silicon nitride, microwave sintering, mechanical properties, cytocompatibility, anti-bacteria

## Abstract

Silicon nitride is a bioceramic with great potential, and multiple studies have demonstrated its biocompatibility and antibacterial properties. In this study, silicon nitride was prepared by a microwave sintering technique that was different from common production methods. SEM and pore distribution analysis revealed the microstructure of microwave-sintered silicon nitride with obvious pores. Mechanical performance analysis shows that microwave sintering can improve the mechanical properties of silicon nitride. The CCK-8 method was used to demonstrate that microwave-sintered silicon nitride has no cytotoxicity and good cytocompatibility. From SEM and CLSM observations, it was observed that there was good adhesion and cross-linking of cells during microwave-sintered silicon nitride, and the morphology of the cytoskeleton was good. Microwave-sintered silicon nitride has been proven to be non-cytotoxic. In addition, the antibacterial ability of microwave-sintered silicon nitride against *Staphylococcus aureus* and *Escherichia coli* was tested, proving that it has a good antibacterial ability similar to the silicon nitride prepared by commonly used processes. Compared with silicon nitride prepared by gas pressure sintering technology, microwave-sintered silicon nitride has excellent performance in mechanical properties, cell compatibility, and antibacterial properties. This indicates its enormous potential as a substitute material for manufacturing bone implants.

## 1. Introduction

Bone tissue has a natural regenerative ability, but its repair function has limitations and cannot meet the repair needs of most injuries [1]. If the damage exceeds the limit of bone tissue repair capacity, bone tissue engineering materials need to be implanted in the damaged location. Every year, millions of bone tissue replacement surgeries are performed using implanted materials worldwide, making bones second only to blood in terms of demand for transplanted materials [2]. The basic requirement for any bone tissue engineering biomaterial is biocompatibility, which is defined as the ability of the material to have appropriate host reactions in a specific application [3]. Bioceramics are a commonly used inorganic material in bone tissue engineering. Bioceramics are divided into bioactive ceramics and biologically inert ceramics. Generally speaking, bioactive ceramics are those that can integrate with surrounding tissues; otherwise, they are considered bioinert ceramics. Common bioactive ceramics include calcium phosphate and bioactive glass, while common bioinert ceramics include alumina and zirconia.

Silicon nitride is a non-oxide ceramic and is considered a ceramic–glass composite material. Silicon nitride ceramics exhibit excellent mechanical properties, characterized by high hardness, high compressive strength, and strong fracture toughness [4,5,6,7]. In addition, silicon nitride also has advantages such as good wear resistance, acid and alkali corrosion resistance, wave transmission, and excellent thermal conductivity [4,8,9]. Based on these advantages, silicon nitride has been widely used in fields such as machinery, semiconductors, and aviation. In recent years, silicon nitride has entered people’s vision as a promising bioceramic. At first, silicon nitride was considered a bioinert ceramic. Numerous studies have shown that silicon nitride has no significant cytotoxicity to cells in vitro [10,11,12]. Silicon nitride was implanted into animals, and there were no adverse reactions in the surrounding tissues [13,14,15]. With the attempted application of silicon nitride to repair bone defects, the osteogenic properties of silicon nitride have also been widely studied. In vitro experiments have shown that silicon nitride can lead to osteoblast differentiation, and in animal experiments, silicon nitride has higher bone adhesion and growth, indicating that silicon nitride has bone conductivity [16,17,18,19]. In addition, previous studies have demonstrated the biocompatibility, bone integration, and bone formation ability of silicon nitride in the human body by implanting it into the human body and conducting a 30-year radiological fusion evaluation [20]. Therefore, in recent years, Pezzotti has believed that silicon nitride should be a bioactive material [21,22]. As an implant material, antibacterial activity is also an important property. Bacteria form biofilms and colonize the surface of materials, which can cause orthopedic infections, leading to implant loosening and bone damage that cannot heal [23,24]. Silicon nitride has been found to have antibacterial effects on Gram-positive and Gram-negative bacteria. A lot of in vitro studies have demonstrated the antibacterial activity of silicon nitride against different microorganisms such as *Escherichia coli*, *Staphylococcus epidermidis*, and *Porphyromonas gingivalis* [23,25,26,27,28,29,30,31,32]. Silicon nitride also exhibits excellent antibacterial properties in animals [33]. Pezzotti et al. proposed the antibacterial mechanism of silicon nitride against Gram-positive bacteria and Gram-negative bacteria, respectively [22,31].

Manufacturing methods such as reaction sintering, gas pressure sintering, and hot pressing sintering have emerged to meet the needs of silicon nitride with different performances [24]. In recent years, microwave sintering has also been applied to the production of silicon nitride. Microwave sintering (MS) is a sintering process that involves coupling materials with microwaves, absorbing electromagnetic energy, generating dielectric losses, and heating both the interior and exterior of ceramic materials simultaneously. Therefore, the material can be uniformly heated by overall heating, achieving densification. Densification gives sintered products better mechanical strength. Compared with other traditional sintering methods, the microwave, as a clean energy source, is more environmentally friendly. In addition, microwave sintering can also shorten sintering time and reduce energy consumption, making it a more economical sintering process [34]. Previous studies have shown that silicon nitride with lithium yttrium oxide and zirconia as sintering additives has been successfully prepared using microwave sintering technology [35]. The fracture toughness and Vickers hardness of this silicon nitride increase with an increase in sintering temperature.

By combining the low energy consumption and improved material mechanical properties of the microwave sintering process, as well as the good biocompatibility of silicon nitride, microwave sintering is used to prepare silicon nitride. This study aimed to preliminarily prove the feasibility of microwave-sintered silicon nitride as an implant material and verify whether microwave sintering technology will affect the mechanical properties, cytotoxicity, and antibacterial properties of silicon nitride. The microstructure, porosity, and mechanical properties of microwave-sintered silicon nitride and gas-pressure-sintered silicon nitride were mainly compared. The cytotoxicity of microwave-sintered silicon nitride was detected and compared with that of gas-pressure-sintered silicon nitride and other control materials. The adhesion and cytoskeleton morphology of cells on microwave-sintered silicon nitride were observed. The antibacterial properties of microwave-sintered silicon nitride were also measured.

## 2. Materials and Methods

### 2.1. Preparation of Materials

Two kinds of silicon nitride used in these studies were produced by Hengyang Kaixin Special Materials Technology Co., Ltd. (Hengyang, China). We used Al_2_O_3_, Y_2_O_3_, and MgO at a total content of 10% as sintering additives for silicon nitride, with a ratio of 2:5:3 for Al_2_O_3_, Y_2_O_3_, and MgO. The mixed powder of silicon nitride and sintering additives was added to the ball milling tank in a 1:1 ratio of anhydrous ethanol. Then, a planetary ball mill was used for 2 h. After drying completely, the mixture was passed through a 40-mesh sieve. Green bodies were formed through isostatic pressing at 200 MPa and placed in a sintering furnace for sintering. The first type of silicon nitride was prepared using microwave sintering (MS), with a microwave frequency of 2.45 GHz. A nitrogen-protective atmosphere was introduced, and the sintering chamber had a positive pressure of 1000 Pa. The heating rate was 5 °C/min, and the sintering temperature was 1600 °C. It was kept for 2 h. Preparation of the second kind of silicon nitride samples used gas pressure sintering (GPS) technology, a sintering temperature of 1690 °C, nitrogen pressure applied at 1.8 MPa, a heating rate of 2 °C/min, and a holding time of 3 h. All silicon nitride samples were polished with diamond grinding wheels. According to GB/T 16886.12 (Biological evaluation of medical devices, Part 12: Sample preparation and reference material, 2005) and ISO 10993-8 (Biological evaluation of medical devices—Part 8: Selection and qualification of reference materials for biological tests, 2000) [36,37], and the recommendations of other authors [11,12,16,23,28,31], alumina ceramic, titanium alloy, and polyetheretherketone (PEEK) were used as references (provided by Hengyang Kaixin Special Materials Technology Co., Ltd.). All materials were made into circular discs with a diameter of 10 mm and a thickness of 3 mm.

### 2.2. Physical Characterization of Materials

SEM (TESCAN Bmo, s.r.o., TESCAN MIRA, Brno, Czech Republic) was used to observe the microstructure and surface of silicon nitride ceramic discs. The pore size and porosity of the samples were analyzed using the mercury intrusion pore method (MIP) (Micromeritics, Autopore IV 9500, Norcross, GA, USA). Under a certain pressure, mercury can penetrate pores, and the pressure is inversely proportional to the size of the pores. Sample phase analysis was performed by XRD (RIGAKU, Smartlab SE, Tokyo, Japan) by using monochromatic Cu-Kα radiation (λ = 1.5406 Å). The wettability of the material was measured using a contact angle tester (Chengde Dingsheng, JY-82C, Chengde, China). Three independent measurements were performed by dropping 3 μL of ultrapure water onto the different positions of the sample surface. The measurement of the surface roughness of materials was completed by the three-dimensional laser microscopic imaging system (Keyence, VK-150K, Osaka, Japan). The density of silicon nitride was measured by the Archimedes drainage method. The Vickers hardness of silicon nitride was measured by using a hardness tester, applying a test force of 98.1 N (10 kg) to the material using an indenter, and holding it for 15 s to obtain hardness data. The flexural strength, compressive strength, and fracture toughness of the material were tested with an electronic universal testing machine.

### 2.3. Cytotoxicity Test

Mouse osteoblast-like MC3T3-E1 cells (Procell Life Science&Technology Co., Ltd. Wuhan, China) were used in the in vitro cell experiment. The MC3T3-E1 cells were cultured with 5% CO_2_ at 37 °C in α-minimum essential medium with 10% (*v*/*v*) fetal bovine serum and 1% penicillin/streptomycin, and the medium was replaced every 3 days. Before cells achieved confluence, 0.25% trypsin EDTA (Procell Life Science&Technology Co., Ltd.) was applied to collect cells. Before inoculating cells into ceramic discs, cells were cultured for 3–5 generations.

The sample discs were sterilized by autoclaving and then placed into the new, sterilized 24-well plates (Saining Biotechnology Co., Ltd., Suzhou, China). The collected cells were resuspended in fresh medium to a density of 3 × 10^4^ cells/mL. Then, 1 mL of cell suspension was added to the well containing sample discs, and they were cultured in an environment with 5% CO_2_ at 37 °C for 24 h. The proliferation of MC3T3-E1 cells on the samples was evaluated using the Cell Counting Kit-8 (Elabscience, Wuhan, China). At a specific time point, the culture medium was removed, and the samples were washed three times with PBS. Then, α-MEM medium containing 10% CCK-8 solution was added to the wells and incubated for 2 h. After culturing for 2 h, 100 μL of the medium was transferred to a 96-well plate for measurement. The OD values were measured by using a microplate reader at a wavelength of 450 nm.

### 2.4. Cell Adhesion and Morphology

MC3T3-E1 cells were seeded on the discs at a density of 4 × 10^4^ cells per well in 24-well plates and cultured for 1 day. The samples were then stained with 2-(4-Amidinophenyl)-6-indolecarbamidine dihydrochloride (DAPI, Beyotime Biotech, Shanghai, China) and Actin-Tracker Red-Rhodamine (Beyotime Biotech, Shanghai, China) to observe the cell morphology using confocal laser scanning microscope (CLSM, Zessi, LSM800, Oberkochen, Germany). The morphology of the adhered cells on the discs was also observed by SEM (TESCAN Bmo, s.r.o., TESCAN MIRA, Brno, Czech Republic) after dehydrating the samples in gradient ethanol solutions (30, 50, 70, 85, 90, and 100%, respectively) for 10 min and drying at the critical point.

### 2.5. Evaluation of Antibacterial Property

Antibacterial properties of materials against *Staphylococcus aureus* and *Escherichia coli* were determined by colony count method. Luria-Bertani broth was used to culture *S. aureus* (ATCC 25923) and *E. coli* (ATCC 25922). The microbiological culture was inoculated with the material and incubated at 37 °C for 24 h. A certain amount of PBS was used to strongly clean the material, causing microorganisms to detach from the material. Sterilized PBS was used to dilute the eluent. A total of 100 μL of diluted eluent was added to the LB broth agar medium. This was then incubated at 37 °C for 24 h, and the number of colonies counted.

### 2.6. Statistical Analysis

Each experiment used three experimental samples to statistically analyze the experimental data. The mean ± standard deviation is used to represent the experimental results. Student *t*-tests and one-way analysis of variance (ANOVA) were performed on the data using statistical product and service solution 26.0 software (SPSS 26.0, IBM, New York, NY, USA); * *p* < 0.05 indicates statistical significance.

## 3. Results

### 3.1. Characterization of Materials

**Figure 1:**
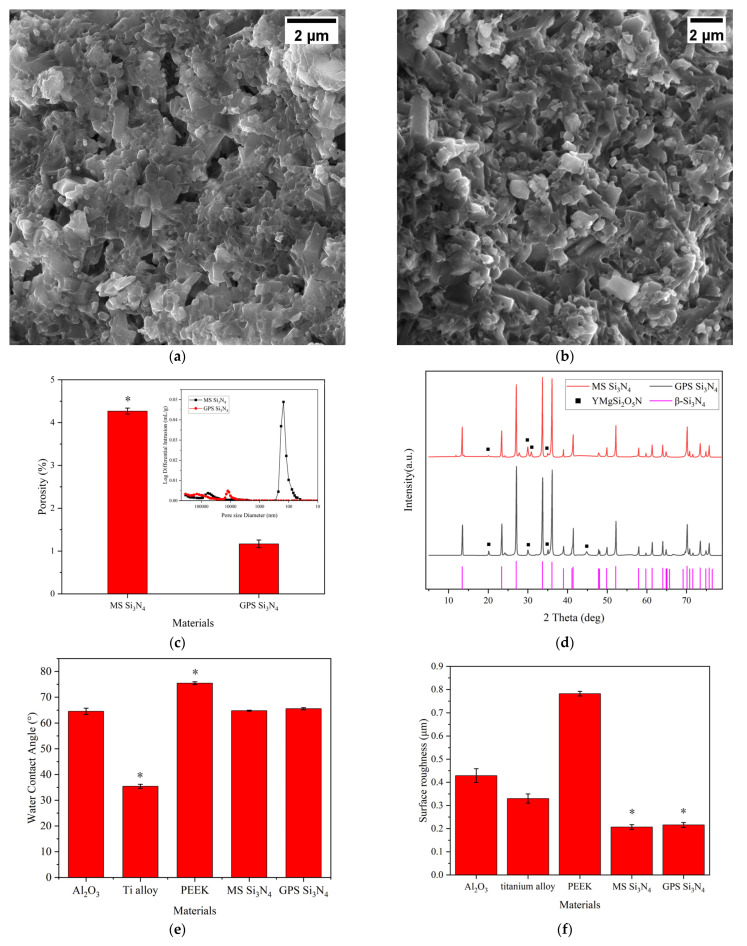
(**a**) SEM image of MS silicon nitride cross-section (magnification factor: 15.00 k×), (**b**) SEM image of GPS silicon nitride cross-section (magnification factor: 15.00 k×), (**c**) Porosity of silicon nitride samples (insert: pore size distribution of MS and GPS silicon nitride samples), (**d**) XRD pattern of silicon nitride samples, (**e**) Wettability of each material, (**f**) Surface roughness of each material (* *p* < 0.05).

The observation of MS and GPS silicon nitride using scanning electron microscopy is shown in Figure 1a,b. It can be clearly observed that there were slender hexagonal prismatic silicon nitride crystals in the cross-section between the two types of silicon nitride. These silicon nitride crystals intersected and exhibited anisotropy. However, there were still significant differences in the cross sections between the two types of silicon nitride. For the MS silicon nitride in Figure 1a, several irregular pores with a cross-sectional size of approximately 1.39 ± 0.31 μm can be observed on its cross-section. On the contrary, compared with MS silicon nitride, it can be clearly observed that the crystals in the GPS silicon nitride cross-section were denser and had no obvious pores.

An estimation of the porosity and pore size distribution of two types of silicon nitride using MIP is shown in Figure 1c. According to MIP, the porosity of GPS silicon nitride and MS silicon nitride was measured (Figure 1c) as an MS silicon nitride porosity of 4.27 ± 0.07%, which was significantly higher than that of GPS silicon nitride (* *p* < 0.05), which was only 1.17 ± 0.09%. The illustration in Figure 1c shows the pore size distribution of two types of silicon nitride, which exhibit significant differences in pore size distribution. According to the red pore size distribution curve in the illustration, the most probable pore size of GPS silicon nitride is 12,492.64 nm, and its median pore size is 79,304.42 nm. Different from that, the black pore size distribution curve shows that the most probable pore size of MS silicon nitride is 150.83 nm, with a median pore size of 160.08 nm. The XRD spectra of two types of silicon nitride are shown in Figure 1d. From Figure 1d, it can be seen that both MS and GPS silicon nitrides form the main phase of β-Si_3_N_4_. The characteristic peaks of β-Si_3_N_4_ (pink solid line) at 2θ = 13.5°, 23.4°, 27.1°, 33.7°, 36.1°, 41.4°, 52.1°, and 70.1° appear in the XRD spectra of both types of silicon nitrides. The secondary phase is YMgSi_2_O_5_N, whose characteristic peaks (black solid square) at 2θ = 20.1°, 24.3°, 27.9°, 30.1°, and 44.9° also appear in the XRD spectra of MS and GPS silicon nitrides. The intensity of the characteristic peaks of YMgSi_2_O_5_N is much lower than that of β-Si_3_N_4_. The wettability results of each material are shown in Figure 1e and Supplementary material Figure S2. The results show the water contact angles of various materials. Obviously, PEEK has the highest water contact angle, reaching 75.44 ± 0.53°. MS silicon nitride and GPS silicon nitride reach 64.82 ± 0.21° and 65.53 ± 0.38°, respectively. The contact angle of alumina is basically the same as that of silicon nitride, at 64.56 ± 1.17°. The titanium alloy is the lowest at 35.44 ± 0.77°. The roughness of the material surface is shown in Figure 1f. Figure S1 showed the surface morphology of the materials, which are supplement to Figure 1f. The surface roughness of two silicon nitride materials was significantly lower than that of the other three control materials, which were 0.207 μm and 0.216 μm, respectively. Their surface morphology is basically the same. Among the control materials, PEEK had the highest surface roughness at 0.782, followed by aluminum oxide at 0.429 μm and titanium alloy at 0.33 μm, which was the lowest. The lower surface roughness of silicon nitrides was due to the polishing of the surface with diamond grinding wheels.

### 3.2. Mechanical Properties of Materials

The mechanical performance parameters of two types of silicon nitride samples were measured, and the results are shown in Table 1. The parameter data for the control material are sourced from [38]. The density and Vickers hardness of the two types of silicon nitride are relatively similar. The density of the GPS silicon nitride sample is 3.207 ± 0.021 g/cm^3^, and the Vickers hardness is as high as 14.73 ± 0.28 GPa. The density of the MS silicon nitride samples is 3.198 ± 0.030 g/cm^3^, and the Vickers hardness is as high as 14.83 ± 0.33 GPa. The density of both types of silicon nitride is lower than that of titanium alloy and alumina ceramic, while the hardness is greater than that of titanium alloy and PEEK. The flexural strength of GPS silicon nitride samples is as high as 523 ± 55 MPa, and the flexural strength of MS silicon nitride samples is as high as 621 ± 63 MPa. The flexural strength of MS silicon nitride is significantly greater than that of GPS silicon nitride, and the performance parameter of both is higher than that of alumina ceramic, which has 3–4 times the bending strength of PEEK. In terms of compressive strength, the GPS silicon nitride reaches 2465 ± 210 MPa, while the MS silicon nitride reaches 2586 ± 265 MPa, which is 2.5–3 times that of titanium alloy and more than 20 times that of PEEK. Only a few alumina ceramics can reach 3000 MPa. In terms of fracture toughness, the GPS silicon nitride is 7.47 ± 0.41 MPa·m^1/2^, which is lower than the 8.12 ± 0.54 MPa·m^1/2^ of MS silicon nitride. The fracture toughness of titanium alloy is 9–10 times that of silicon nitride, and that of alumina ceramic is lower, at 4–5 MPa·m^1/2^.

### 3.3. Cytotoxicity Determination

**Figure 2:**
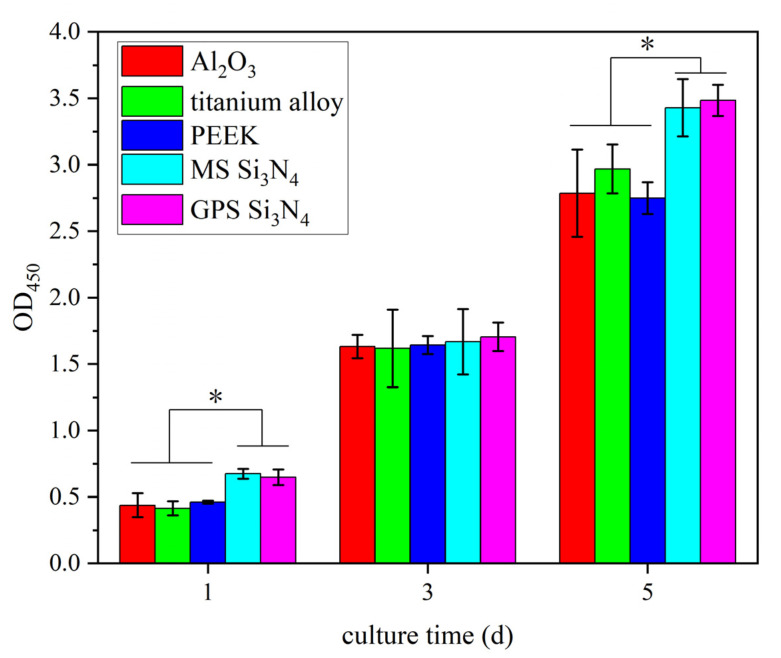
OD value of MC3T3-E1 cells culturing on the samples for different times (* *p* < 0.05).

In vitro cytotoxicity experiments were conducted on MS silicon nitride and control materials to detect their cytotoxicity. The cytotoxic results of MS silicon nitride and other control materials are shown in Figure 2. We used the CCK-8 method to detect cell growth on different materials on days 1, 3, and 5. The cell OD_450_ of all samples increased over time. On the first day, the OD_450_ of both types of silicon nitride was higher than that of the control material (* *p* < 0.05), but there was no significant difference between the two types of silicon nitride. On the third day, the OD_450_ of several materials was significantly increased, indicating that cells in all materials proliferated. On the fifth day, the OD_450_ of both silicon nitride samples was significantly higher than that of other control materials (* *p* < 0.05), indicating that the silicon nitride samples had the highest number of live cells. MS silicon nitride is as non-toxic as GPS silicon nitride.

### 3.4. Cell Adhesion and Morphology

**Figure 3:**
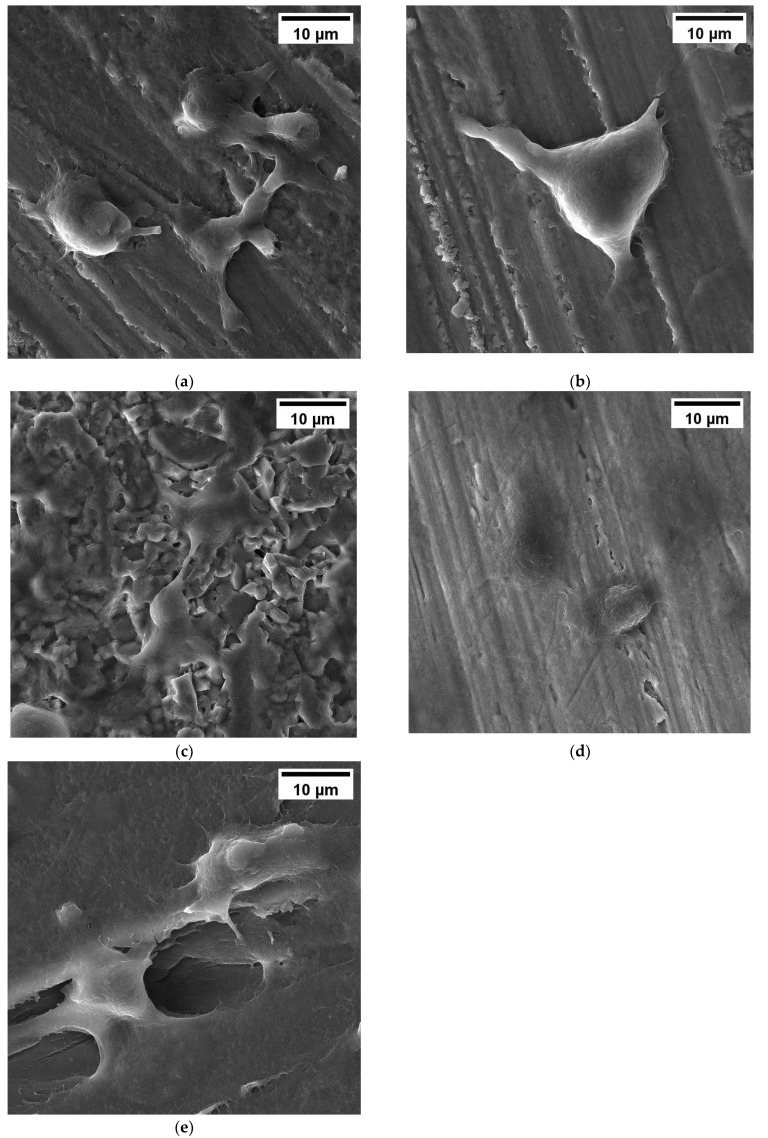
SEM images of MC3T3-E1 cell morphology on the silicon nitrides and control materials. (**a**) MS silicon nitride, (**b**) GPS silicon nitride, (**c**) Alumina ceramic, (**d**) Titanium alloys, (**e**) PEEK. All SEM images have a magnification of 4.00 k×.

Figure 3 shows SEM photos of cells cultured on different materials. In Figure 3, it can be observed that cells can normally adhere to the surfaces of MS silicon nitride and other control materials. The surface of each material is partially covered by cells. The prominent filamentous pseudopodia on the surfaces of silicon nitrides, PEEK, and alumina ceramic are more pronounced, and the cells exhibit typical spindle or irregular shapes. On the contrary, the cells on the surface of titanium alloys are mainly circular. The adhesion of cells on the surface of MS silicon nitride is consistent with that on GPS silicon nitride.

**Figure 4:**
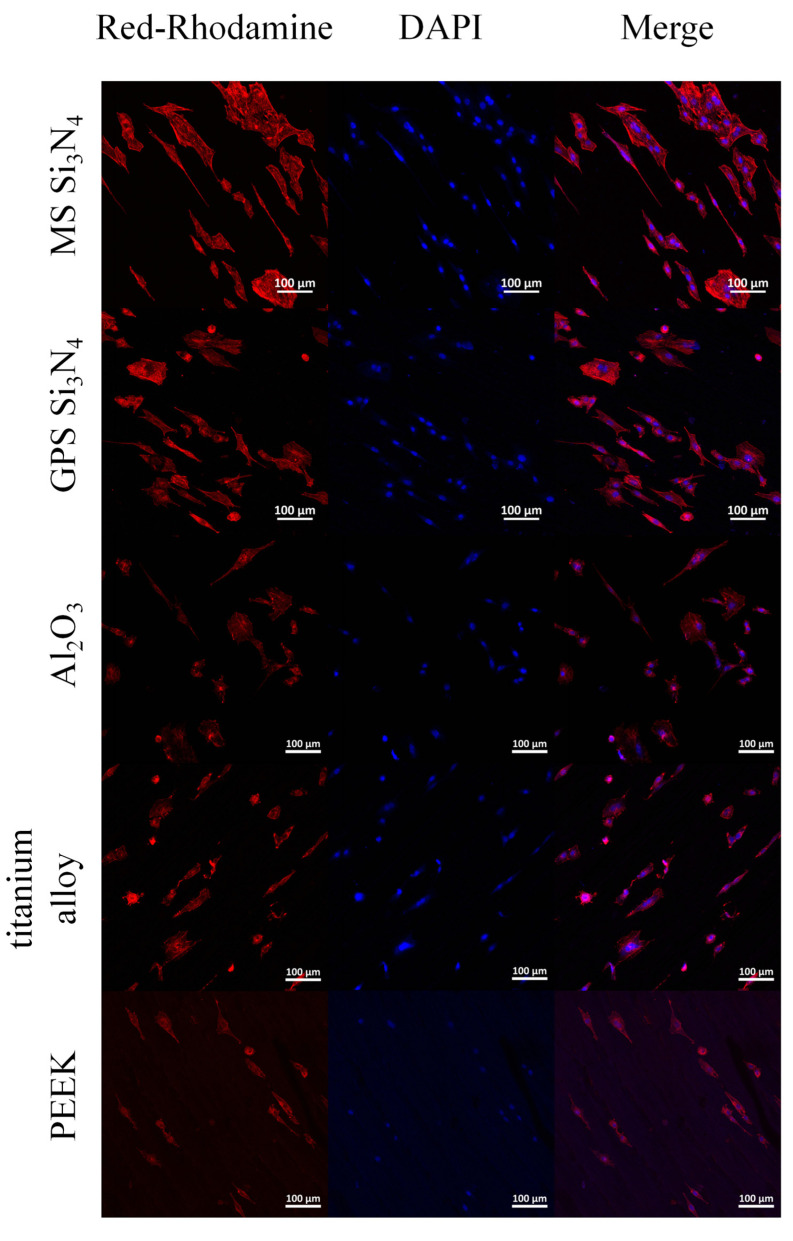
CLSM images of MC3T3-E1 cell cytoskeleton morphology on the silicon nitrides and control materials.

MC3T3-E1 cells attached to the sample were stained using DAPI and Actin Tracker Red Rhodamine and observed using a confocal laser scanning microscope. The results are shown in Figure 4. The effects of different samples on the morphology of the cytoskeleton were observed. The nuclei are blue and the microfilaments are red. Figure 4 shows the cytoskeleton of MS and GPS silicon nitride. It can be clearly observed that there are more nuclei on the surface of both types of silicon nitride. The adhesion of cells on MS silicon nitride is relatively consistent with that on GPS silicon nitride. There are a large number of actin microfilaments in each cell, forming well-shaped and evenly distributed cytoskeletons. Moreover, the cells on MS silicon nitride undergo significant cross-linking through the cytoskeletons.

### 3.5. Evaluation of Antibacterial Property

**Figure 5:**
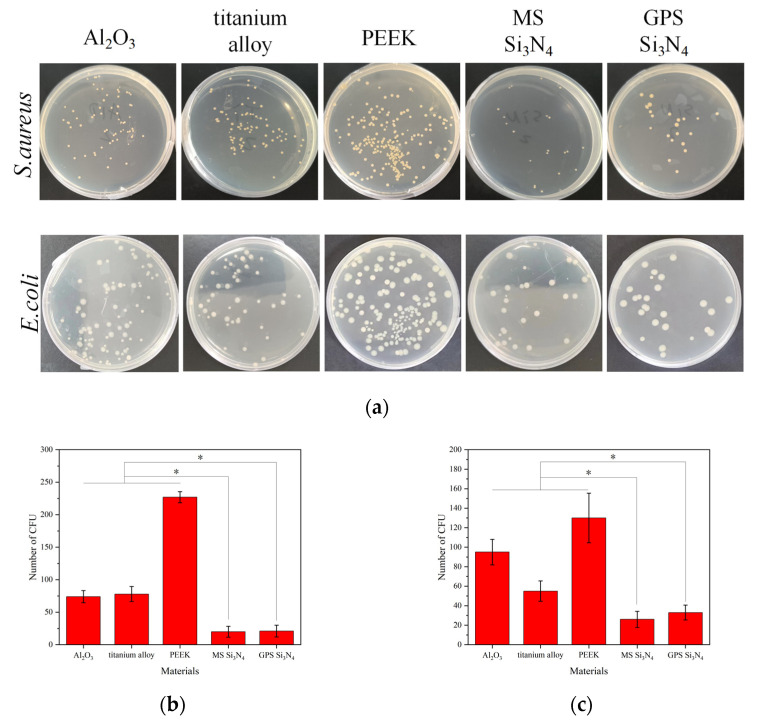
Antibacterial properties of different samples. (**a**) Photographs of *S. aureus* and *E. coli* colonies on the agar plates, (**b**) Comparison of the number of CFUs on different materials of *S. aureus*, (**c**) Comparison of the number of CFUs on different materials of *E. coli* (* *p* < 0.05).

The main reason for inflammation around an implant is bacterial colonization on the surface of the implant, leading to bacterial infection. The use of antibiotics is a commonly used treatment method, and in severe cases, the implant needs to be removed. The ideal implant material should be able to resist bacterial adhesion and proliferation on its surface. Figure 5a shows the cultivation of *S. aureus* and *E. coli* on agar plates after being inoculated on the material and eluted. After inoculation, *S. aureus* and *E. coli* had the highest number of colonies on PEEK culture plates and the lowest number of colonies on the two types of silicon nitride culture plates. The colony-forming units of *S. aureus* on alumina ceramic, titanium alloy, PEEK, GPS silicon nitride, and MS silicon nitride were 74 ± 9.3, 78 ± 11.5, 227 ± 8.6, 20 ± 8.4, and 21 ± 9.1. The colony-forming units of *E. coli* on alumina ceramic, titanium alloy, PEEK, GPS silicon nitride, and MS silicon nitride were 95 ± 13.1, 55 ± 10.4, 130 ± 25.5, 26 ± 8.2, and 33 ± 7.6. The statistics on the number of colony-forming units are shown in Figure 5b,c. The colony counts of MS silicon nitride and GPS silicon nitride were significantly lower than those of other control materials. In Figure 5b,c, * indicates significance (*p* < 0.05).

## 4. Discussion

In this study, silicon nitride prepared by microwave sintering was introduced. We compared the porosity and mechanical properties of silicon nitride prepared by different processes. Biocompatibility is an important measure of implant quality. Therefore, we also tested the cytotoxicity and impact on cell morphology of ceramics. The ability to resist microorganisms is also an essential performance element. Therefore, we also measured the resistance of silicon nitride to *S. aureus* and *E. coli*, which are common in implant infection cases.

Gas pressure sintering is a common method for preparing densified silicon nitride. In contrast, microwave sintering is a new sintering process that involves coupling materials with microwaves, absorbing electromagnetic energy, generating dielectric losses, and heating both the interior and exterior of ceramic materials simultaneously. Microwave sintering is different from conventional sintering in that it can reduce sintering temperature, shorten insulation time, and achieve ceramic densification [34]. The porosity of silicon nitride prepared by both processes in this study is relatively low, and the SEM image in Figure 1a indicates that both types of silicon nitride belong to dense silicon nitride. However, the difference in porosity between the two images may be due to the differences in sintering temperature, sintering time, and gas pressure between the two sintering methods. Within a certain temperature range, the porosity of silicon nitride is inversely proportional to the sintering temperature [39]. As the sintering temperature increases, α-Si_3_N_4_ transforms into β-Si_3_N_4_. During this process, fine particles decrease, coarse particles increase, macropores disappear, and porosity decreases. The sintering temperature of microwave sintering is lower than that of gas pressure sintering, which may lead to an increase in the porosity of MS silicon nitride. Although both sintering methods for silicon nitride use nitrogen gas, the pressure acting on them is different. The purpose of microwave sintering by introducing nitrogen gas to maintain 1000 Pa is to protect the silicon nitride material from high-temperature oxidation and inhibit the decomposition of silicon nitride in an environment of 1600 °C [40,41,42]. The nitrogen pressure used in gas pressure sintering is as high as 1.8 MPa, which is aimed at promoting the densification of silicon nitride. The sintering time also has a certain impact on the densification of the material. Prolonged sintering time will increase densification. Within a certain time range, prolonging the sintering time will promote densification. The sintering time of GPS silicon nitride is longer than that of MS silicon nitride, and the difference in sintering time also leads to differences in the densification of the two silicon nitride materials. However, based on the constant sintering temperature and sample densification conditions, excessive pressure and a long sintering time will cause abnormal growth of silicon nitride crystals, resulting in reduced mechanical properties. Although the pore size shown in Figure 1a is larger than that measured by MIP, the two are not in conflict. There may be a certain difference between the estimated aperture size in SEM images and the aperture size measured by MIP. MIP measures the overall pore size distribution of the sample and represents the pore size distribution of the sample before pressure damage. On the contrary, SEM measures the pore size distribution of small areas in the sample, and there may be some error in the SEM scale if the magnification is too high. This results in limited comparability between the two techniques. The change in porosity will inevitably lead to differences in the mechanical properties of materials. According to Table 1, the mechanical properties of two types of silicon nitride were compared. The Vickers hardness, compressive strength, bending strength, and fracture toughness of MS silicon nitride were significantly improved. Figure 1c shows that the maximum and median pore sizes of GPS silicon nitride are 100 times larger than those of MS silicon nitride, and the pore size also affects the strength of the material. The traditional view is that porosity increases and mechanical strength decreases. Interestingly, according to Table 1 and Figure 1c, the porosity of MS silicon nitride is higher, but the mechanical strength is also improved. This seems to contradict traditional views, but in reality, it does not. Macropores can serve as a source of material fracture, reducing the bending strength and fracture toughness of the material [43]. There are macropores inside GPS silicon nitride, which reduce its mechanical strength. In addition, although the porosity of MS silicon nitride slightly increases, the pore size decreases sharply, making the material denser and resulting in an increase in its mechanical strength. This provides a good physical basis for microwave sintering of silicon nitride as a transplant material. The XRD results in Figure 1d indicate the presence of YMgSi_2_O_5_N in the sample. YMgSi_2_O_5_N is the main crystalline phase of the oxygen–nitrogen glass–ceramics formed during the sintering process of silicon nitride. Single silicon nitride powder cannot achieve high densification through gas pressure or hot pressure sintering. Adding sintering additives can solve this problem. Sintering additives such as MgO, Al_2_O_3_, and Y_2_O_3_ form eutectic liquid phases with Si_3_N_4_ and its surface SiO_2_ under high-temperature sintering conditions [44,45]. This liquid phase promotes the transformation of α-Si_3_N_4_ into β-Si_3_N_4_ during the solution–reprecipitation process of the silicon nitride sintering process [46,47]. After the liquid phase cools down, an oxygen–nitrogen glass phase forms between the silicon nitride crystals, achieving the densification of silicon nitride.

The CCK-8 kit was used to detect the cytotoxicity of two types of silicon nitride, proving that MS silicon nitride also has no cytotoxicity and that the number of live cells on the surface of MS silicon nitride is greater than that of other control materials. Neuman et al. measured the cytotoxicity of five industrial standard silicon nitride materials and compared them with other control materials [15]. The results indicate that the industrial standard silicon nitrides have no cytotoxicity, and the number of live cells on some silicon nitride is also higher than other control materials. Cappi et al. reported that hot-pressed and pressureless sintered silicon nitride is non-toxic to hMSC cells and promotes osteogenic differentiation [12]. Si and N are the main elements of silicon nitride. The rapid growth of cells on silicon nitride may be related to these two elements. Kim et al. found that Si ions can promote the proliferation, differentiation, and expression of osteoblast markers [48]. Pezzotti et al. demonstrated for the first time that Si and N elements can simultaneously stimulate the differentiation of osteosarcoma and mesenchymal cells, as well as the activity of osteoblasts [22]. The N element of silicon nitride is generally dissolved in the form of NH_4_^+^ under steady-state or neutral pH conditions, which is metabolized as a nutrient through the glutamine synthase pathway. Glutamine is an important energy source that provides the necessary C and N elements for the synthesis of amino acids, nucleotides, glutathione, and hexosamine. The surface roughness of the material also affects cell yield. Some in vitro studies emphasize that surface polishing of silicon nitride can improve cell yield and proliferation ability. It is reported that the polished surface is more suitable for bone cell growth. This results in higher cell and osteocalcin production [16,49,50].

In the reaction process of cells to biomaterials, the first thing that occurs is cell adhesion, which affects the diffusion and proliferation of cells on the surface of the material [51]. Osteoblasts use integrin to adhere to materials, and the expression of integrin is influenced by the surface properties of the material, such as surface roughness, microstructure, and wettability [52]. In this study, SEM images showed that cells on both types of silicon nitride exhibited good adhesion and diffusion states, with more pseudopodia. Compared with other control materials, MS silicon nitride exhibits better adhesion and diffusion morphology, with more obvious pseudopodia. From the CLSM image, it can be observed that the cells exhibit a distinct spindle shape on both types of silicon nitride. Some cells are elongated or even cross-linked, reflecting a clear proliferation process. This collectively indicates that MS silicon nitride has a certain promoting effect on cell proliferation and adhesion.

Bacteria are prone to adhere to the surface of implants, colonizing them to form bacterial biofilms, which in turn cause inflammatory reactions and lead to implant failure [23]. Figure 5 shows that MS silicon nitride, like gas-pressure-sintered silicon nitride, exhibits excellent antibacterial activity against *Staphylococcus aureus* and *Escherichia coli*. Not only bacterial strains but also the surface morphology, wettability, and chemical environment of materials can affect bacterial adhesion [28]. In this study, similar to the commonly used process of silicon nitride, MS silicon nitride also exhibits good antibacterial properties against *S. aureus* and *E. coli*. The antibacterial properties of silicon nitride are attributed to multiple factors. Surface roughness is one of the factors affecting the antibacterial properties of materials. Generally speaking, rough surfaces have more pores and cracks, which provide a location for bacteria to adhere. Therefore, a smooth and non-porous surface is less susceptible to bacterial adhesion [53]. The surface roughness of MS silicon nitride is one of the lowest of all materials. This reduces the opportunity for bacteria to adhere to silicon nitride materials. Moreover, in terms of wettability, the two types of silicon nitride are superior to PEEK but inferior to titanium alloy. The hydrophilic surface of the material will inhibit the adhesion of bacteria to hydrophobic surfaces. However, surface roughness and wettability do not play a dominant role in affecting bacterial adhesion. In addition to surface roughness and wettability, the chemical reactions on the surface of silicon nitride in liquid environments also have a significant impact on its antibacterial properties. Silicon nitride reacts with water in an aqueous environment to produce Si-OH groups on its surface. Previous studies have reported that Si-OH groups can undergo dissociation, causing negative charges to be carried on the surface of silicon nitride [28]. The bacterial surface is a net negative surface, thus inhibiting bacterial adhesion to the surface of silicon nitride through electrostatic repulsion [54]. In addition, Pezzotti et al. found that active nitrogen species (RNS) were formed on the surface of silicon nitride in physiological pH environments, causing damage to the DNA/RNA of Gram-positive bacteria [22]. On the contrary, for Gram-negative bacteria, silicon nitride causes bacterial stress to promote the metabolic degradation of nucleic acids and nucleotide-containing molecules to form uric acid, while NH_3_ and NH_4_^+^ accumulate in the bacteria to form osmotic stress, ultimately leading to bacterial lysis. The change in pH can also affect bacterial adhesion. Some studies have reported that silicon nitride can dissolve NH_3_ in aqueous environments and increase the environmental pH from 4.5 to 8.5 [26]. Weak alkaline environments are not conducive to bacterial adhesion. In this study, it was observed that *S. aureus* and *E. coli* died on the surfaces of two types of silicon nitride. According to the existing antibacterial theory of silicon nitride, the main reason for this phenomenon may be the chemical reaction that occurs on the surface of silicon nitride in the water environment, but further research is still needed to clarify.

This study has demonstrated the preliminary advantages of MS silicon nitride as a bioceramic material for bone therapy in terms of its mechanical properties, cell compatibility, and antibacterial properties. The MS silicon nitride has been proven to be non-cytotoxic and beneficial for cell adhesion. Future in vivo experiments will focus on the impact of microwave-sintered silicon nitride on animal tissues, especially bone tissue. Microwave-sintered silicon nitride as a bioceramic will continue to be deeply studied, further optimizing the production process, improving performance, and laying the foundation for its future application as a transplant in orthopedic surgery.

## 5. Conclusions

This study mainly validated the effects of microwave sintering technology on the mechanical strength, cytotoxicity, and antibacterial properties of silicon nitride. The microstructure and porosity of microwave sintering and pressure sintering of silicon nitride have been detected. Mechanical performance tests have proven that microwave sintering can improve the mechanical strength of silicon nitride. Microwave-sintered silicon nitride has no cytotoxicity, nor does gas-pressure-sintered silicon nitride, and it also improves cell survival rates and provides a suitable location for cell adhesion. MC3T3-E1 cells exhibit excellent adhesion and proliferation on microwave-sintered silicon nitride. It has been found that microwave-sintered silicon nitride also has significant antibacterial activity against *S. aureus* and *E. coli*. Good mechanical properties, no cytotoxicity, and good antibacterial ability indicate that microwave-sintered silicon nitride is a very promising implant material. However, further in-depth research is needed on the osteogenic characteristics of microwave-sintered silicon nitride and its impact on animal physiological activities. The future direction of this work is to combine in vitro and in vivo experiments with clinical trial data, improve the preparation process of silicon nitride, and manufacture high-performance microwave-sintered silicon nitride implants.

## Figures and Tables

**Table 1:** Mechanical properties of two types of silicon nitride and control materials.

Materials	Density (g/cm^3^)	Vickers Hardness (GPa)	Flexural Strength (MPa)	Compressive Strength (MPa)	Fracture Toughness (MPa·m^1/2^)
MS Si_3_N_4_	3.198 ± 0.030	14.83 ± 0.33	621 ± 63	2586 ± 265	8.12 ± 0.54
GPS Si_3_N_4_	3.207 ± 0.021	14.73 ± 0.28	523 ± 55	2465 ± 210	7.47 ± 0.41
Titanium alloy [38]	4.43	3.4	/	950–990	75
Alumina ceramic [38]	3.986	14–16	300–500	2000–3000	4–5
PEEK [38]	1.29	/	160–180	130–140	/

## Data Availability

The data supporting the findings of this study are available from the corresponding author upon reasonable request.

## Supplementary Materials

The following supporting information can be downloaded at: https://www.mdpi.com/article/10.3390/jfb14110552/s1, Figure S1: SEM images of materials surface; Figure S2: Wettability images of each material.
