# Supplementary material for: Silicon Nitride Bioceramics Sintered by Microwave Exhibit Excellent Mechanical Properties, Cytocompatibility In Vitro, and Anti-Bacterial Properties
Source: J Funct Biomater. 2023 Nov 17;14(11):552. doi: 10.3390/jfb14110552 (PMC10671902; doi:10.3390/jfb14110552)
Supplement: Supplementary file 1 [file jfb-14-00552-s001.zip › jfb-2697278-supplementary.pdf]

Journal of *Functional Biomaterials*

MDPI

Supplementary Materials

Silicon Nitride Bioceramics Sintered by Microwave Exhibit Excellent Mechanical Properties, Cytocompatibility *In Vitro*, and Anti-Bacterial Properties

Jiayu He [1], Yuandong Liu [1,*], Xiaofeng Zeng [2,3], Yan Tong [1], Run Liu [1], Kan Wang [1], Xiangdong Shangguan [1], Guanzhou Qiu [1] and Coswald Stephen Sipaut [3]

[1] Key Laboratory of Biohydrometallurgy of Ministry of Education, School of Minerals Processing and Bioengineering, Central South University, Changsha 410083, China; 215611005@csu.edu.cn (J.H.); 235612137@csu.edu.cn (Y.T.); 202208600021012@ctgu.edu.cn (R.L.); 225611016@csu.edu.cn (K.W.); 215612085@csu.edu.cn (X.S.); qgz@mail.csu.edu.cn (G.Q.)

[2] Hengyang Kaixin Special Material Technology Co., Ltd., Hengyang 421200, China; jishuyfdept@hykaixin.com

[3] Faculty of Engineering, University Malaysia Sabah, 88400 Kota Kinabalu, Malaysia; css@ums.edu.my

* Correspondence: yuandong_liu@csu.edu.cn; Tel.: +86-731-88877472

Citation: He, J.; Liu, Y.; Zeng, X.; Tong, Y.; Liu, R.; Wang, K.; Shangguan, X.; Qiu, G.; Sipaut, C.S. Silicon Nitride Bioceramics Sintered by Microwave Exhibit Excellent Mechanical Properties, Cytocompatibility In Vitro, and Anti-Bacterial Properties. *J. Funct. Biomater.* **2023**, *14*, x. https://doi.org/10.3390/xxxxx

Academic Editor(s): **

Received: 25 October 2023
Revised: 13 November 2023
Accepted: 15 November 2023
Published: date

Copyright: © 2023 by the authors. Submitted for possible open access publication under the terms and conditions of the Creative Commons Attribution (CC BY) license (https://creativecommons.org/licenses/by/4.0/).

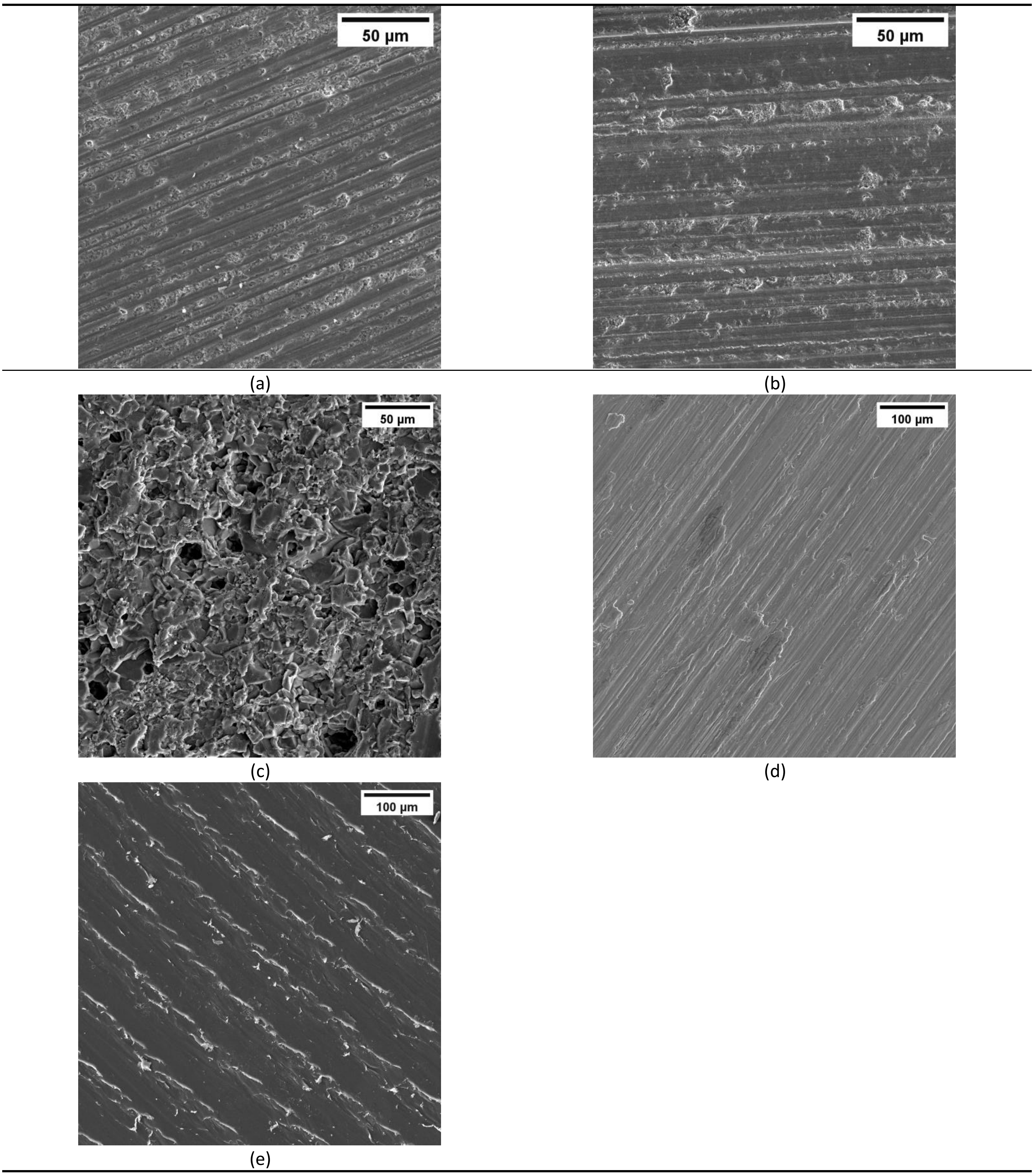

Figure S1. SEM images of materials surface (**a**: MS silicon nitride, **b**: GPS silicon nitride, **c**: alumina ceramic, **d**: titanium alloy, **e**: PEEK).

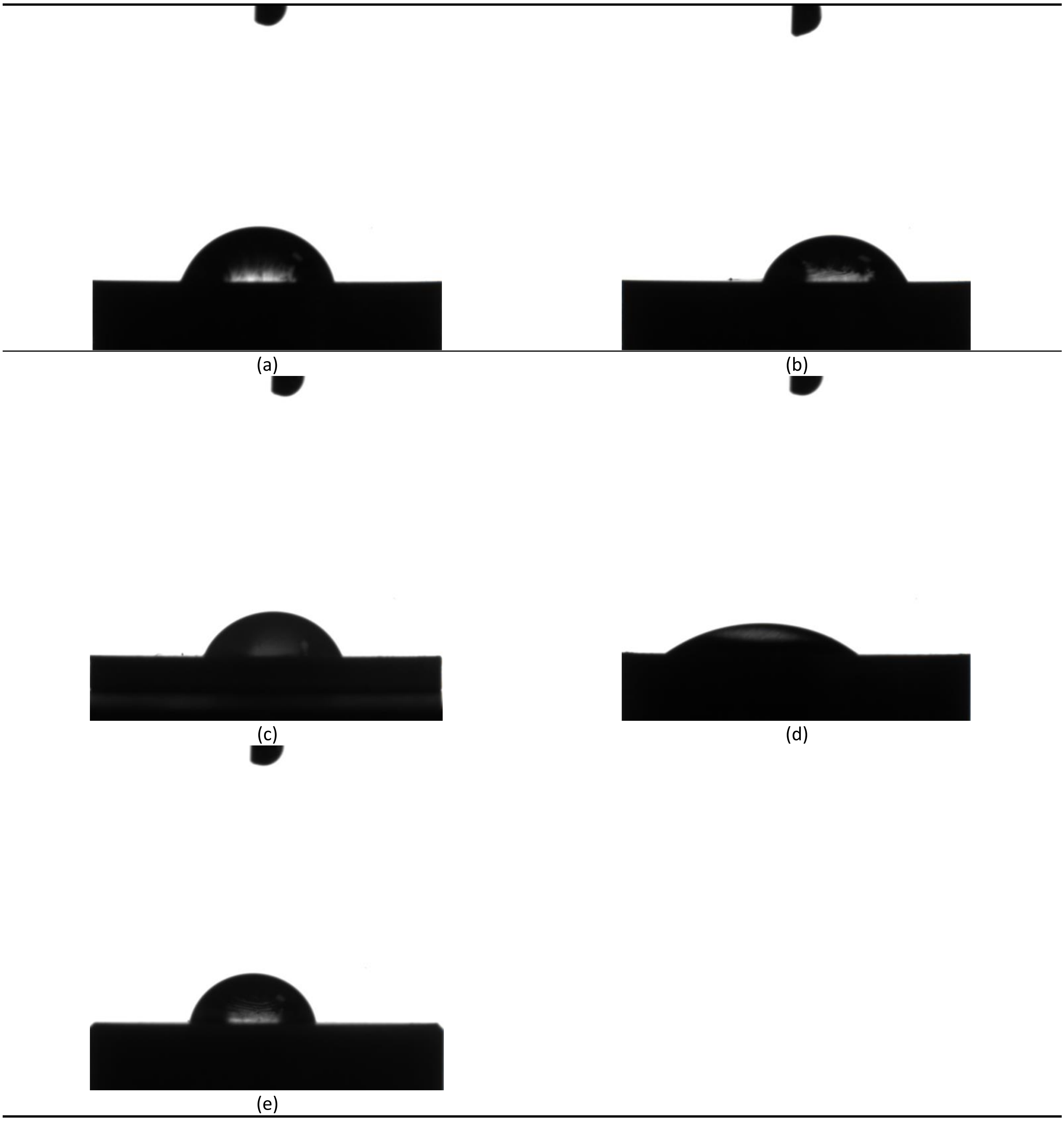

Figure S2. Wettability images of each material (**a**: MS silicon nitride, **b**: GPS silicon nitride, **c**: alumina ceramic, **d**: titanium alloy, **e**: PEEK).
